# Do Serum 25-Hydroxyvitamin D Concentrations Affect Body Composition, Physical Fitness, Bone Strength and Bone Biomarkers in Female Children and Adolescent Football Players? A One-Season Study

**DOI:** 10.3390/ijerph192215394

**Published:** 2022-11-21

**Authors:** Gabriel Lozano-Berges, Ángel Matute-Llorente, Alejandro Gómez-Bruton, Alex González-Agüero, Germán Vicente-Rodríguez, José A. Casajús

**Affiliations:** 1 GENUD (Growth, Exercise, NUtrition and Development) Research Group, Universidad de Zaragoza, 50009 Zaragoza, Spain; 2 Faculty of Health and Sport Sciences (FCSD), Department of Physiatry and Nursing, Universidad de Zaragoza, 22001 Huesca, Spain; 3 Instituto Agroalimentario de Aragón (IA2), Universidad de Zaragoza, 50009 Zaragoza, Spain; 4 Centro de Investigación Biomédica en Red de Fisiopatología de la Obesidad y Nutrición (CIBEROBN), 28029 Madrid, Spain; 5 Faculty of Health Sciences (FCSD), Department of Physiatry and Nursing, Universidad de Zaragoza, 50009 Zaragoza, Spain

**Keywords:** body composition, bone health, fitness, team sport, youth

## Abstract

The aim was to compare changes in body composition, physical fitness, and bone biomarkers in female children and adolescent football players with different Vitamin D levels. Twenty-two players were classified into two groups according to 25(OH)D concentrations: 11 with deficient/insufficient 25(OH)D levels (IVD; <30 ng/mL) and 11 with sufficient 25(OH)D levels (SVD; ≥30 ng/mL). Body composition parameters were measured using dual-energy X-ray absorptiometry and a peripheral quantitative computed tomography scanner. The following physical fitness tests were applied: maximal isometric knee extension (MIF), long jump, 30-m sprint, and 20-m shuttle run test (VO_2max_). Electrochemiluminescence immunoassays were used to analyze bone biomarkers and 25(OH)D. All variables were registered at the beginning and the end of the football season. The increase in subtotal bone mineral density (BMD) was higher in players with SVD than those with IVD (*p* = 0.030). Only players with SVD improved their MIF of the left leg (*p* = 0.005); whereas, only players with IVD decreased their 30-m sprint performance (*p* = 0.007) and VO_2max_ (*p* = 0.046). No significant between- and within-group differences were found for bone biomarkers. SVD might cause an extra improvement of subtotal BMD in female children and adolescent football players. Moreover, it seems that the 25(OH)D concentration could be an important parameter for physical fitness improvement in this population.

## 1. Introduction

Vitamin D (Vit D) regulates calcium levels and bone mineralization and has recently been shown to be a steroid hormone constituting a true hormonal axis with systemic and local actions in different cells, organs, and systems, in fact, its deficiency could increase the risk of osteoporosis, autoimmune disorders, or cancer, among others [1]. The Endocrine Society defines Vit D deficiency as blood levels of 25-hydroxyvitamin D (25(OH)D) below 20 ng/mL (50 nmol/L) and recommends 25(OH)D concentrations above 30 ng/mL (75 nmol/L) to maximize its effects on bone development and calcium absorption [1]. A systematic review performed by Manios et al. [2] observed a high prevalence of low Vit D concentrations in Southern Europe during childhood and adolescence, critical periods of bone development. Thus, presenting adequate Vit D levels during growth may reduce a person’s risk for bone health problems.

There is evidence suggesting that Vit D has a positive relationship with cardiorespiratory fitness, skeletal muscle development, and at the same time, muscle strength and performance [3,4,5,6]. For instance, a systematic review and meta-analysis performed by Tomlinson et al. [7] reported positive effects of Vit D supplementation on strength of upper and lower limbs in young adults with Vit D deficiency (mean of 25(OH)D serum levels: 12.3 ng/mL). Additionally, this vitamin may have an important function in skeletal muscle remodeling after fatiguing exercises, which is clearly related to athletic performance [8]. Nevertheless, these effects have been observed only in participants with levels of Vit D below the recommendation (<30 ng/mL) being inconclusive in those with Vit D levels above the recommendation. 

Previous authors evaluated seasonal variations in Vit D concentrations and their associations with bone and muscle health in different sports (football, basketball, volleyball, and swimming, among others); nevertheless, the majority of these studies were performed in adult male athletes [9,10]. In female adolescent football players, a study performed by Brännström et al. [11] evaluated the relationship between Vit D and bone health and muscle strength/power parameters finding only a moderate correlation with knee extension peak torque. Despite the fact that the positive association between these parameters might prevent future diseases such as osteoporosis, the evidence in this population remains scarce and to our knowledge, there are few studies that evaluate the influence of Vit D status on bone and muscle performance changes in female athletes. Therefore, the present study aimed to evaluate changes in body composition, physical fitness, and bone biomarkers in children and adolescent female football players taking into account their Vit D concentration. We hypothesized that the increase in the previously commented parameters would be higher in female players with sufficient 25(OH)D levels (SVD) compared to those with deficient or insufficient 25(OH)D levels (IVD).

## 2. Materials and Methods

### 2.1. Participants

An initial sample of 33 female football players from three football clubs in Zaragoza (Spain) agreed to participate in this study. Eleven players were excluded for the following reasons: five football players refused blood tests, and six did not attend the day when physical performance tests were performed. Consequently, the final sample of the present study consisted of 22 female football players (mean age: 12.6 ± 0.6 years old). Participants were divided into the following two groups according to their 25(OH)D concentrations: IVD (25(OH)D < 30 ng/mL) or SVD (25(OH)D ≥ 30 ng/mL). Twelve of these players had experienced menarche before the beginning of the study (mean age: 12.8 ± 0.6 years old; eight players were in the IVD group (total of 11 players) and four in the SVD group (total of 11 players). No proportion differences between football players with IVD and SVD in pre- and post-menarche groups were found (χ^2^(1) = 2.291, *p* = 0.130).

Although football exercises performed during practices were not the same among teams, the structure was similar and comparable. Practices lasted approximately 90 min, including a 5-min warm-up consisting of low-intensity running; 5–10 min of low-intensity games; 60 min of technical football exercises (passing, kicking, running, and dribbling); and 5–10 min of cool-down stretching exercises. A sport scientist registered the attendance at practices and monitored the type of exercises performed by each team. This made it possible to quantify the total hours of training per week individually.

This study is a part of a randomized controlled trial registered in a public database Clinicaltrials.gov [NCT02399553]. The research followed the Declaration of Helsinki 1961 (revision of Fortaleza 2013) and was approved by the Ethics Committee of Clinical Research from the Government of Aragon (CEICA, Spain; PI13/0091). Participants, parents, and coaches were informed of the protocol of this study and its benefits and risks. After that, all participants gave verbal consent, and their parents signed written informed consent. 

### 2.2. Inclusion Criteria

Participants had to be Caucasian, between 11 and 14 years old, play football for at least one year before being involved in the study, and be free of any medication that could affect bone properties or development.

### 2.3. Assessments

All the assessments described below were carried out on two occasions. The first evaluation took place at the beginning of the season (October–December; Autumn) while the second assessment was conducted during the final part of the season (May–July; Spring–Summer). Measurements took place in the GENUDLab located in Zaragoza (Spain).

#### 2.3.1. Anthropometric Measurements

Height (stadiometer SECA 225, SECA, Hamburg, Germany; to the nearest 0.1 cm) and weight (scale SECA 861, SECA, Hamburg, Germany; to the nearest 0.1 kg) were evaluated without shoes and the minimum clothes. Body mass index (BMI) was calculated by dividing weight (kg) by squared height (m^2^).

#### 2.3.2. Maturity Offset and Age of Peak Height Velocity

Maturity offset was calculated using the equation developed by Moore et al. [12]:

Maturity offset = −7.709133 + (0.0042232 × (age × height in cm)).

The age of peak height velocity was calculated by subtracting chronological age from maturity offset.

#### 2.3.3. Body Composition

Dual-energy X-ray absorptiometry (DXA) QDR-Explorer (pediatric version of the software QDR-Explorer, Hologic Corp., Software version 12.4, Bedford, MA, USA) was used to measure subtotal (total body less head) bone mineral density (BMD; g/cm^2^), subtotal lean mass (kg) and percentage of body fat (%), all obtained from whole-body scans. The same technician performed all scans and daily calibrations following the manufacturer’s guidelines. Coefficients of variation of whole-body BMD and lean mass in our laboratory are already published, being 1.3 and 1.9%, respectively [13]. 

Bone strength index (BSI was calculated as total area multiplied by squared total density; mg/mm) [14] at the 4% site of the non-dominant tibia and polar strength strain index (SSIp; mm^3^) at the 38% site of the non-dominant tibia were measured using a Stratec XCT-2000 L peripheral quantitative computed tomography scanner (pQCT; Stratec Medizintechnik, Pforzheim, Germany). Leg dominance was determined by asking which leg would be used to kick a ball [15]. After that, participants were seated on an adjustable chair. Then, the tibial length was determined from the medial knee joint cleft to the medial malleolus of the tibia using a wooden ruler. The scanner was positioned on the distal tibia, and a scout view was performed to manually set the reference line on the midpoint of the distal tibia endplate. The same technician performed all scans and calibrated pQCT equipment using a quality control phantom provided by the manufacturer. Coefficients of variation of pQCT variables in our laboratory are already published [16], being less than 5.3%.

#### 2.3.4. Physical Fitness

Maximum isometric knee extension force (MIF; kg) was measured using a strain gauge in both legs (MuscleLab, Force Sensor, Stathelle, Norway). Participants sat on a table with an anchorage placed on the distal tibia. They were instructed to perform the highest knee extension force for approximately 4–6 s. Each player had three attempts per leg with at least 3-min rest between the same leg attempts. The best attempt of MIF in each leg was retained for the statistical analysis. 

Maximum running velocity (s) during 30 m was calculated using two pairs of photocells (Byomedic fotoelectric cells, Barcelona, Spain) placed at the starting (0 m) and finishing (30 m) lines. Participants performed three maximal attempts with a minimum rest of 3-min between them and only the best time was selected for the statistical analysis. 

Maximal dynamic strength was evaluated with the Long Jump test. Players had to jump as far as possible, taking off and landing with both feet at the same time. After landing, a researcher measured the length (m) between the starting line and the closest heel. Three attempts with at least 3-min rest between them were allowed. The longest jump was selected.

Maximal oxygen uptake (VO_2max_) was estimated by the 20-m shuttle run test (Course Navette). Participants ran between two lines separated by 20-m at a speed determined by audio signals emitted from a pre-recorded compact disc with an initial velocity of 8.0 km/h and increases of 0.5 km/h per min. The test stopped if the participant did not reach the line for two consecutive beeps. Finally, age (years) and the velocity reached (km/h) were used to estimate VO_2max_ using the Leger’s equation [17]:

VO_2max_ = 31.025 + (3.238 × velocity) − (3.248 × age) + (0.1536 × velocity × age)

#### 2.3.5. Bone Biomarkers and Biochemical Parameters

Fasting blood samples were extracted from all participants between 8:00 and 10:00 a.m., were centrifuged at 10.000 rpm for 15 min, and were clotted at room temperature. Serum samples were separated and stored at −80 °C. Serum 25(OH)D, procollagen type I N-terminal propeptide (P1NP), osteocalcin (OC), and C-terminal cross-linked telopeptide (CTX) were evaluated by electrochemiluminescence immunoassays (ECLIA), using an Elecsys 2010 analyzer (Roche Diagnostics, GmbH, Grenzach-Wyhlen, Germany). Furthermore, the OC-CTX ratio was calculated by dividing OC by CTX. The intra-assay coefficients of variation for 25(OH)D ranged from 4.3% to 6.4%, for P1NP ranged from 2.6% to 3.0%, for OC ranged from 1.8% to 3.8%, and for CTX ranged from 2.1% to 4.9%. Calcium in serum was measured by colorimetric assay; its coefficients of variation ranged from 1.4% to 1.6%. All analyses were performed by an accredited medical laboratory (Centro inmunológico de Alicante-CIALAB, Alicante, Spain).

### 2.4. Statistical Analysis

The sample size calculation was performed for the ANOVA for repeated measures, the main analysis of this study. The sample size for repeated measures was calculated in subtotal body BMD to get a power of 90% at the 5% alpha level and to reject the null hypothesis H_0_: μ1 = μ2. Thus, assuming a small-to-medium effect size (f = 0.20) and a correlation among repeated measures of 0.9 at pre- and post-season, a total sample size of 16 (8 per group) would be needed. G*Power analysis program was used to compute sample size calculation and statistical power analyses [18].

Statistical Package for the Social Sciences (SPSS) version 24.0 for MacOS (SPSS Inc., Chicago, IL, USA) was used for all statistical analyses. Data are presented as mean (standard deviation) for normally distributed variables, and median (interquartile range) for those showing no normal distribution. The significance level was set at 0.05. The Shapiro–Wilk test was used to analyze the distribution of the variables. Training years, training hours, BSI, MIF of right leg, sprint velocity, long jump, CTX, 25(OH)D variables did not show normal distribution.

Differences between players with IVD and SVD for descriptive characteristics and primary variables at the beginning and the end of the season were analyzed using independent *t*-tests for normally distributed variables. Mann–Whitney U tests were used for those that did not show a normal distribution. Analysis of variance (ANOVA) for repeated measures was used to check differences between players with IVD and SVD between the beginning and the end of the season for normally distributed variables. Wilcoxon signed-rank test was performed for those that did not show a normal distribution. Group-by-time interactions could only be applied for normally distributed variables. 

Effect size statistics using Cohen’s d were calculated for the independent t-test, partial eta square (η^2^_p_) for repeated measures analyses, and effect size r for Mann–Whitney U and Wilcoxon signed-rank tests. The effect size for Cohen’s d can be small (0.2–0.5), medium (0.5–0.8), or large (0.8); η^2^_p_ can be small (0.01–0.06), medium (0.06–0.14), or large (>0.14); and r can be small (0.1–0.3), medium (0.3–0.5), or large (>0.5).

## 3. Results

The descriptive characteristics of the included participants are shown in Table 1. No significant differences between IVD and SVD groups were found in any descriptive variable neither at the beginning nor the end of the season (Cohen’s d ranged from 0.021 to 0.359 and r for training years and training hours were 0.092 and 0.197, respectively; *p* > 0.05). Both IVD and SVD groups significantly increased their age, weight, height, and maturity offset along the season (η^2^_p_ ranged from 0.293 to 0.930; *p* < 0.05).

Body composition, physical fitness, and bone biomarkers of football players with IVD and SVD are shown in Table 2. There were no differences between groups for the other evaluated variables (Cohen’s d ranged from 0.00 to 0.47 and r ranged from 0.03 to 0.38; *p* < 0.05). Both groups significantly increased subtotal BMD, subtotal lean mass, MIF of the left leg, and long jump (η^2^_p_ ranged from 0.223 to 0.779 and r were 0.49 to 0.60, respectively; *p* < 0.05; statistical power ranged from 0.417 to 1.000), and decreased calcium levels (η^2^_p_ were 0.454 and 0.463, respectively; *p* < 0.05; statistical power = 0.999) from the beginning to the end of the season. Additionally, SVD players decreased body fat percentage (η^2^_p_ was 0.202; *p* = 0.036; statistical power = 0.852) and serum 25(OH)D levels (r was 0.57; *p* = 0.005; statistical power = 0.923), and increased MIF of the right leg (r was 0.57; *p* = 0.005; statistical power = 0.642). Sprint velocity (r was 0.55; *p* = 0.007; statistical power = 0.994) and VO_2max_ (η^2^_p_ was 0.184; *p* = 0.046; statistical power = 0.810) decreased in participants with IVD from the beginning to the end of the season. Group-by-time interactions were found for subtotal BMD (η^2^_p_ was 0.214; *p* = 0.030; statistical power = 0.607). This interaction demonstrated that the increase in subtotal BMD was higher in football players with SVD compared to those with IVD.

## 4. Discussion

The main finding of the present study is that sufficient levels of 25(OH)D seem to provoke an extra improvement of subtotal BMD in children and adolescent female football players even though both SVD and IVD groups significantly increased subtotal BMD between the beginning and the end of the season. This extra increase in participants with SVD could be explained by the fact that high serum 25(OH)D levels during adolescence induce intestinal absorption of calcium and phosphorus [1]. Besides, together with the osteogenic effect of football practice [19], they may achieve greater bone development, although this affirmation cannot be confirmed by the lack of a control group. No significant serum calcium differences were found between SVD and IVD groups. This lack of differences could be justified by the fact that serum calcium levels may be regulated by Vit D or parathyroid hormone [1]. It has been observed that Vit D deficiency altered calcium absorption and bone metabolism, and at the same time, increased parathyroid hormone levels [1]. In a Vit D deficient environment, this increase of parathyroid hormone maintains adequate calcium levels through increasing osteoclastic activity although this process weakens bones [1,20]. Thus, the calcium data of this study should be cautiously interpreted due to the lack of parathyroid hormone control in the present project and the presence of only one player of the IVD group with Vit D deficiency.

In contrast with subtotal BMD findings, no significant differences were found between Vit D groups in bone strength parameters measured at the non-dominant tibia. It should be noted that both SVD and IVD groups were exposed to specific football loads, which cause bone micro damage and increase bone remodeling activity [21]. This remodeling induces bone adaptations and changes in shape and geometry to sustain higher impacts [22] which could partially explain the absence of significant bone strength differences between Vit D groups. In addition to this, Bano et al. [23] reported no associations between serum 25(OH)D levels and bone strength parameters in young adults. Therefore, sufficient Vit D concentrations might not cause an extra improvement in bone strength parameters as was observed in subtotal BMD.

There were also no bone biomarker differences between the groups. Bone biomarkers provide more dynamic information on bone formation and resorption activity [24] without directly reflecting the amount of bone gained during growth [25]. As described above, bone metabolism is altered when serum 25(OH)D levels are below 20 ng/mL [1] and our sample only had one football player of the IVD group with Vit D deficiency (25(OH)D = 19.2 ng/mL). Thus, bone biomarkers of the majority of the sample seem not to be affected by Vit D concentrations. 

Vit D has also been related to skeletal muscle and physical performance [3]; nevertheless, this association has been mainly studied in healthy adolescents [6], adults [7], older adults [26], or elite athletes [10,27]. In adolescent athletes, a previous study including female football players did not find any correlations between Vit D and skeletal muscle and performance except for peak torque in knee extension, which showed a moderate correlation with Vit D [11]. In line with these results, in the present study, only players with SVD improved the MIF of the right leg. Regarding the other physical fitness parameters, only the IVD group decreased the 30-m sprint performance and VO_2max_ from the beginning to the end of the season without observing these decreases in the SVD group. Despite these physical fitness differences, it should be highlighted that both SVD and IVD groups increased their subtotal lean mass which could be a growth-related change. Based on these results, there seems to be a positive association between 25(OH)D and muscle health. In fact, there is evidence demonstrating the role of Vit D on calcium uptake in muscle cells [28] and the presence of Vit D receptors in them [29], which might have effects on muscle contraction. 

The main limitation of the present study is the lack of Vit D intake and parathyroid hormone levels. They are relevant parameters to understand the effects of Vit D on bone and muscle health, as stated herein [1]. Another limitation is the absence of a control group that would allow observing the effects of sports practice on bone, in combination with Vit D levels. Moreover, participants’ experience in terms of training years and training hours per week could be too low to affect the possible influence of different Vit D levels on the parameters studied. On the other hand, it should be highlighted that this study analyzes how different Vit D concentrations may affect bone and muscle parameters during the football season. Additionally, these effects have been evaluated in adolescent female players when most studies include adult males [7,9,10].

## 5. Conclusions

The present study demonstrates that presenting a sufficient level of serum 25(OH)D concentration might induce extra BMD acquisition in children and adolescent female football players. Besides that, there seems to be better physical fitness in football players with SVD compared to those with IVD. Nevertheless, these results should be cautiously interpreted due to the fact that no group-by-time interactions were found for physical fitness parameters, and the absence of a control group is an important limitation that makes it more difficult to obtain a causal effect of different Vit D levels on body composition, physical fitness, and bone biomarker parameters in this population. Therefore, future studies which include a higher sample of participants and a control group are recommended to address these limitations.

Based on the results obtained in the present study, we would recommend children and adolescent female football players increase their Vit D levels through an adequate intake of Vit D-rich foods and sufficient exposure to natural sunlight in order to improve their body composition and physical fitness parameters.

## Figures and Tables

**Table 1:** Descriptive characteristics of female football players with different 25(OH)D concentrations.

		IVD	SVD		Effect Size
Variables		N = 11	N = 11	*p*-Value	d or r
Age	T0	12.6 (0.6)	12.6 (0.6)	0.907	0.051
(years)	T1	13.2 (0.6) #	13.3 (0.6) #	0.654	0.194
Weight	T0	47.5 (7.6)	50.0 (10.9)	0.542	0.264
(kg)	T1	50.5 (7.9) #	52.5 (10.6) #	0.623	0.213
Height	T0	154.9 (5.7)	154.7 (8.0)	0.925	0.041
(cm)	T1	157.6 (4.9) #	158.6 (7.4) #	0.707	0.163
BMI	T0	19.7 (2.2)	20.8 (3.5)	0.410	0.359
(kg/m^2^)	T1	20.3 (2.6)	20.8 (3.3)	0.704	0.164
Maturity offset	T0	0.5 (0.4)	0.5 (0.8)	0.933	0.036
(years)	T1	1.1 (0.4) #	1.2 (0.8) #	0.577	0.242
Age at PHV	T0	12.1 (0.4)	12.1 (0.3)	0.961	0.021
(year)	T1	12.1 (0.4)	12.1 (0.3)	0.793	0.114
Training years ^1^	T0	1 (1–7)	1 (1–6)	0.688	0.092
(years)	T1	-	-	-	-
Training hours ^1^	T0	2.7 (1.9–3.4)	3.14 (2.1–3.4)	0.371	0.197
(h/week)	T1	-	-	-	-

Data are presented as mean (standard deviation) for normally distributed variables, and median (interquartile range) for those showing no normal distribution. Abbreviations: T0: beginning of the season; T1: end of the season; IVD: football players with deficient or insufficient 25(OH)D concentration group; SVD: football players with sufficient 25(OH)D concentration group; BMI: body mass index; PHV: peak height velocity; d: Cohen’s d; r: effect size r. ^1^ These variables were not normally distributed. # Significant differences within groups between the beginning and the final of the season.

**Table 2:** Body composition, physical performance, bone biomarkers and blood biochemical parameters of female football players with different 25(OH)D concentrations.

					Within		Group by Time Interaction
					IVD	SVD	
		IVD	SVD	Effect Size	*p*-Value	*p*-Value	*p*-Value
Variables		N = 11	N = 11	d or r	(η^2^_p_ or r)	(η^2^_p_ or r)	(η^2^_p_)
Body composition							
SB BMD	T0	0.854 (0.071)	0.854 (0.080)	0.003	**<0.001**	**<0.001**	**0.030**
(g/cm^2^)	T1	0.895 (0.074)	0.921 (0.074)	0.352	(0.565)	(0.779)	(0.214)
SB lean mass	T0	30.5 (4.6)	32.1 (6.1)	0.292	**0.002**	**<0.001**	0.259
(kg)	T1	32.4 (4.1)	34.9 (6.2)	0.471	(0.381)	(0.570)	(0.063)
Body fat	T0	27.4 (4.2)	27.4 (6.9)	0.290	0.818	**0.036**	0.094
(%)	T1	27.6 (5.3)	25.4 (4.7)	0.445	(0.003)	(0.202)	(0.134)
BSI	T0	101.2 (19.5)	94.5 (26.4)	0.290	0.416	0.100	0.535
(mg/mm)	T1	103.5 (20.9)	99.2 (29.0)	0.170	(0.033)	(0.129)	(0.020)
SSIp	T0	1226.4 (219.7)	1143.0 (256.0)	0.350	0.134	0.186	0.855
(mm^3^)	T1	1240.8 (277.4)	1194.1 (188.9)	0.197	(0.114)	(0.090)	(0.002)
Physical performance							
MIF-R ^1^	T0	37.3 (35.5–40.3)	40.8 (33.6–45.0)	0.091	0.328	**0.008**	
(kg)	T1	39.4 (36.0–43.5)	45.8 (37.9–53.4)	0.329	(0.209)	(0.569)	-
MIF-L	T0	35.7 (5.9)	38.7 (7.0)	0.462	**0.027**	**0.023**	0.961
(kg)	T1	39.5 (5.7)	42.6 (10.3)	0.374	(0.223)	(0.233)	(0.000)
Sprint 30 m ^1^	T0	5.5 (5.2–5.6)	5.5 (5.3–5.6)	0.063	**0.010**	0.059	
(s)	T1	5.2 (5.1–5.4)	5.3 (5.1–5.5)	0.379	(0.550)	(0.402)	-
Long Jump ^1^	T0	1.4 (1.4–1.6)	1.5 (1.4–1.6)	0.168	**0.005**	**0.020**	
(m)	T1	1.5 (1.4–1.7)	1.5 (1.5–1.6)	0.098	(0.600)	(0.494)	-
VO_2max_(CN)	T0	46.1 (2.4)	44.7 (5.1)	0.367	**0.046**	0.426	0.364
(mL/kg·min)	T1	43.0 (4.8)	43.5 (5.4)	0.098	(0.184)	(0.032)	(0.041)
Bone biomarkers							
P1NP	T0	579.9 (330.1)	646.8 (235.5)	0.233	0.125	0.178	0.888
(μg/mL)	T1	452.5 (260.3)	535.5 (250.1)	0.325	(0.113)	(0.089)	(0.001)
OC	T0	127.7 (42.3)	124.7 (40.8)	0.073	0.251	0.250	0.998
(μg/mL)	T1	113.3 (52.2)	110.2 (42.7)	0.065	(0.065)	(0.066)	(0.000)
CTX ^1^	T0	1.4 (1.1–2.6)	1.8 (1.7–2.1)	0.063	1.000	0.008	
(μg/mL)	T1	1.3 (1.2–2.4)	1.5 (1.2–2.0)	0.035	(0.000)	(0.152)	-
OC/CTX	T0	75.0 (21.0)	72.5 (10.8)	0.154	0.114	0.478	0.518
(ratio)	T1	66.0 (15.9)	68.5 (13.6)	0.169	(0.120)	(0.026)	(0.021)
Biochemical parameters	T0						
25(OH)D ^1^	T0	24.9 (24.8–25.8) $	40.2 (34.3–42.5)	0.848	0.423	**0.008**	
(ng/mL)	T1	22.9 (20.4–28.6) $	30.6 (27.1–36.3)	0.581	(0.171)	(0.569)	-
Calcium	T0	10.1 (0.3)	10.2 (0.2)	0.144	**<0.001**	**0.001**	0.959
(mg/dL)	T1	9.6 (0.3)	9.6 (0.3)	0.160	(0.463)	(0.454)	(0.000)

Data are presented as mean (standard deviation) for normally distributed variables, and median (interquartile range) for those showing no normal distribution. Abbreviations: T0: beginning of the season; T1: end of the season; IVD: football players with deficient or insufficient 25(OH)D concentration group; SVD: football players with sufficient 25(OH)D concentration group; SB: subtotal body; BMD: bone mineral density; BSI: bone strength index at the 4% site of the tibia; SSIp: polar strength strain index at the 38% site of the tibia; MIF-R: maximum isometric knee extension force in right leg; MIF-L: maximum isometric knee extension force in left leg; VO_2max_(CN): maximal oxygen uptake obtained from Course Navette Test; P1NP: Procollagen type I N-terminal propeptide; OC: Osteocalcin; CTX: C-terminal cross-linked telopeptide; OC/CTX: osteocalcin and C-terminal cross-linked telopeptide ratio; 25(OH)D: serum 25-Hydroxyvitamin D concentration; d: Cohen’s d; *p*: significance level; r: effect size r; η^2^_p_: partial eta square. ^1^ These variables were not normally distributed. $ Significant differences when compared to SVD. Significant differences within groups between the beginning and the final of the season and significant group-by-time interactions were shown by *p*-value in bold. Significant level was set at 0.05.

## Data Availability

Not applicable.
